# A Simple Method of Overcoming a Difficulty

**Published:** 1857-06

**Authors:** R. Arthur


### A SIMPLE METHOD OF OVERCOMING A DIFFICULTY.

In filling teeth, a very difficult part of the operation is to keep the moisture away from the cavity. There are some cases, in which the secretion of saliva is so great, that it is impossible to prevent the cavity from being overflowed, and we are obliged to do the best we can under the circumstances; these are extreme cases, however, by making use of the right means, and with care, we are usually able to keep the saliva from interfering with the operation. But where the cavity to be filled is situated on the proximate surface of a tooth, near the gum, the great obstacle in the way of a good operation, when "annealed gold" is used, is the secretion of mucus. In some cases, too, the gum projects slightly into the cavity, and in the course of preparation, may have been slightly wounded so as to bleed easily.

Several methods have been suggested to overcome this difficulty. A wedge of wood, in cases where it is admissible, is sometimes thrust between the teeth, so as to force the gum out of the way, and so compress it as to stop, for the time, the secretion of mucus, and the bleeding. Where this cannot be done, the gum is sometimes touched with nitrate of silver, or chloride of zinc, and the natural action of the vessels suspended for the time. Dr. Jack proposed, and practiced, he states, with satisfaction, packing a very small piece of bibulous paper under the tree edge of the gum.

A very simple method has lately suggested itself to me, which I have put into practice with so much satisfaction that I communicate it. I have at hand a number of the india-rubber tubes used for regulating the teeth, of several sizes. From one of these I cut off a small ring, taking the sized tube suitable for the case in hand. This ring is passed around the tooth to be filled, embracing it alone or several others if necessary; it may be brought up against the gum with so much force as to press it out of the way, if it projects into the cavity, and to compress it so as completely to stop the mucus secretion, or any slight bleeding. The use of this appliance for the purpose proposed is so simple as to require no further explanation. It is only necessary to try it to be convinced of its value.—*News Letter.* R. ARTHUR.

